# From basic to clinical translation: advances and perspectives of photodynamic nanodrugs

**DOI:** 10.3389/fphar.2025.1606372

**Published:** 2025-05-20

**Authors:** Shitang Ma, Shasha Shi, Xin Hu, Ye Zhao, Boran Yang, Maoliang Liao, Baowei Lu, Qilin Xu

**Affiliations:** ^1^ College of Biological and Pharmaceutical Engineering, West Anhui University, Lu’an, China; ^2^ Department of Biochemistry and Molecular Genetics, University of Colorado Anschutz Medical Campus, Aurora, CO, United States; ^3^ Division of Transplantation Immunology, National Research Institute for Child Health and Development, Tokyo, Japan; ^4^ Department of Public Health, International College, Krirk University, Bangkok, Thailand; ^5^ Tianjin Wutong High School, Tianjin, China

**Keywords:** photodynamic nanodrugs, photosensitizer, nanocarriers, multifunctional nanoplatform, immunotherapy, synergistic effect, personalized strategy

## Abstract

Photodynamic nanodrugs (PDNS) have demonstrated significant advantages in enhancing therapeutic outcomes while reducing systemic toxicity, achieved primarily through optimized photosensitizer solubility, targeted biodistribution, and site-specific accumulation. This review systematically examines recent progress and future directions of PDNS development, encompassing fundamental research to clinical translation. Specifically, it analyzes the composition, mechanisms of action, inherent advantages, clinical applications, as well as the challenges faced in this domain. The introduction of nanocarriers has circumvented the limitations of the core photosensitizers, substantially enhancing the efficacy and safety of PDNS via targeted delivery and synergistic therapy. Moreover, the integration of stimuli-responsive and multifunctional nanoplatforms has further improved the spatiotemporal control of reactive oxygen species (ROS) generation, thereby minimizing off-target effects. In addition, the combination of PDNS with immunotherapy has exhibited synergistic effects, underscoring the potential of this integrated approach. PDNS has made remarkable progress in cancer treatment through receptor-mediated endocytosis, self-assembly, and precise targeting. Beyond cancer treatment, PDNS holds considerable promise in treating a diverse array of non-oncological diseases, such as acne, psoriasis, dry eye disease, and cardiovascular disorders, et al. In this regard, PDNS has emerged as a pivotal component within the realm of personalized medicine. Despite these notable advancements, challenges persist in optimizing drug delivery and achieving efficient clinical translation. Looking ahead, future perspectives encompass the development of highly efficient photosensitizers and ensuring accurate nanocarrier delivery, which will undoubtedly facilitate the progress of PDNS in the clinical application field.

## Graphical Abstract

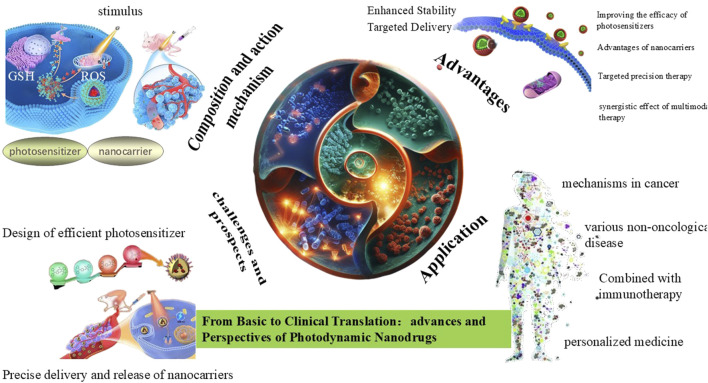

## 1 Introduction

Photodynamic nanodrugs (PDNS), sophisticated nano systems integrating therapeutic photosensitizers with engineered nanocarriers, have emerged as a critical therapeutic modality in modern medicine for both cancer and non-cancer diseases (Wang et al., 2023a). Upon exposure to specific light stimulation, these nanoscale agents trigger the production of reactive oxygen species (ROS), facilitating precise destruction of diseased cells and ultimately achieving therapeutic efficacy. This integrated strategy offers high selectivity and minimal toxicity. For instance, nanocarriers like liposomes and polymer-based systems exhibit varied biocompatibility profiles, and some inorganic PDNS including gold nanoparticles may induce long-term accumulation concerns (Jakic et al., 2024). It overcome early photosensitizer limitations such as inadequate water solubility, suboptimal biodistribution, and insufficient targeting owing to advancements in materials science and medicine (Sarbadhikary et al., 2022).

The evolution of photosensitizers has significantly enhanced the multifunctionality of PDNS, enabling simultaneous imaging and therapeutic capabilities (Li et al., 2022a). Furthermore, innovations in nanomaterials have improved the efficiency of ROS generation and cellular uptake, ensuring precise targeting while minimizing collateral damage (Qi et al., 2019). The incorporation of stimuli-responsive elements has further advanced the field, allowing for controlled photosensitizer release, which further optimizes therapeutic outcomes while maintaining biocompatibility and safety in clinical applications (Naser et al., 2024).

In practice, the development of matched nanocarriers has revolutionized the application of PDNS by enhancing the physicochemical properties and pharmacokinetic behavior of photosensitizers (Marques et al., 2023). These carriers improve water solubility and stability, facilitate selective accumulation via the enhanced permeability and retention (EPR) effect, and enable precise spatiotemporal control through multifunctional designs, such as surface modification and stimulus-responsive release (Islam et al., 2021). Recent innovations include the development of novel photosensitizers and the optimization of metal-organic frameworks (MOFs) and polymer nanoparticles, which exhibit exceptional drug-loading capacity and photothermal conversion efficiency. These systems can be activated by near-infrared (NIR) light, enabling deep tissue penetration (Zhu et al., 2022). Additionally, the incorporation of herbal medicine into PDNS, such as berberine-loaded polymeric nanoparticles, has demonstrated promising synergistic effects, leading to more accurate and safer treatments (Marques et al., 2023; Sulaiman et al., 2022).

Despite significant theoretical and experimental advancements, challenges remain in optimizing drug delivery systems of PDNS for enhanced targeting and penetration in clinical applications. Future research must focus on bridging the gap between laboratory research and clinical translation. This review summarizes recent progress in the development of PDNS, emphasizing its composition, mechanisms of action, and clinical potential. The purpose is to highlight the present advancements and future perspectives in modern therapeutic applications.

### 1.1 Composition and action mechanism of PDNS

The composition and action mechanism are of paramount importance in precisely targeting diseased cells, efficiently generating reactive oxygen species (ROS) to disrupt its structure and function, thereby enhancing therapeutic efficacy while minimizing damage to normal tissues (Chang et al., 2023). Photosensitizers, as the core components, upon light activation, are capable of generating ROS such as singlet oxygen and hydrogen peroxide. These ROS can induce apoptosis or necrosis of target cells by damaging its membranes, DNA, and proteins. Classical photosensitizers include porphyrins and platinum - based polypyrroles (Li et al., 2023b). Although platinum - based polypyrroles have specific applications, they are less well - recognized compared to the porphyrins (German et al., 2024).

**FIGURE 1:**
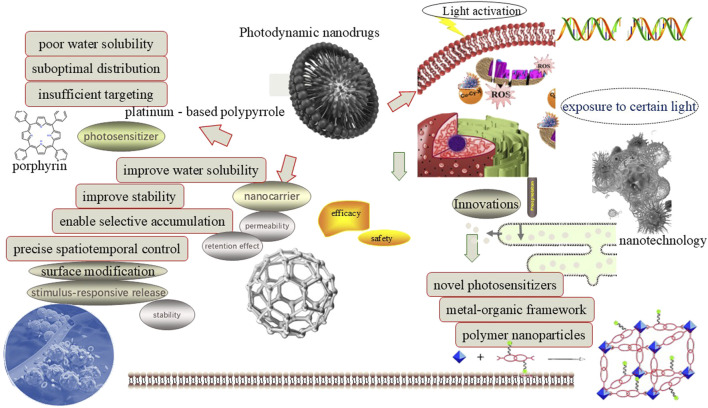
Composition and its innovation for PDNS.

However, these photosensitizers suffer from several limitations in clinics, including low photostability, poor water solubility, and limited tissue penetration depth (Ding et al., 2024). These drawbacks lead to uneven distribution of photosensitizers in tissues and a reduction in the overall efficacy. Nanocarriers are another essential component. It can enhance the stability and bioavailability of its encapsulated photosensitizers by protecting from degradation. Existing nanocarrier materials include inorganic nanoparticles, liposomes, exosomes, and red blood cell membranes (Li et al., 2023a). These nanocarriers can improve the stability of drugs and enable targeted delivery through surface modifications, thus enhancing the therapeutic selectivity and effectiveness of PDNS. Some comparative studies highlight trade-offs between carrier types. For example, liposomes exhibit rapid clearance compared to exosome-based systems, which show prolonged circulation but face scalability challenges (Islam et al., 2021; Marques et al., 2023). Similarly, porphyrins dominate clinically due to strong ROS generation, but emerging NIR agents like ICG offer deeper tissue penetration despite lower photostability (Zhu et al., 2022). Similar to photosensitizers, these nanocarriers also face several limitations in practice. These include potential biocompatibility issues, rapid clearance from the human body leading to short durations of therapeutic effect, as well as nonspecific binding and aggregation in complex biological environments (Mesquita et al., 2024), which may affect its final precise delivery efficiency and therapeutic application (Figure 1).

To address the aforementioned issues of PDNS, numerous strategies have been meticulously explored. Among them, the incorporation of nanotechnology has proven to be an exceptionally effective solution, significantly enhancing drug stability, augmenting targeting efficiency, and ultimately, substantially improving therapeutic outcomes. In the sphere of cancer therapy, bacterium-based PDNS are prepared utilizing lysozyme degradation, ammonium chloride lysis, and nano-extrusion techniques to enhance their efficacy (Gholami et al., 2024). Bacterial membrane-coated nanoparticles (e.g., *P. gingivalis*) enhance tumor targeting via pathogen-associated molecular patterns in OSCC (Shi et al., 2023).

In practice, these PDNS not only exhibit robust therapeutic capabilities but also amplify the effects through immune activation (Liu et al., 2021). Furthermore, it can efficiently encapsulate commonly used chemotherapeutic agents, such as doxorubicin (DOX), through straightforward incubation methods, thereby enabling synergistic effects between PDNS-based immunotherapy and chemotherapy to suppress the growth and metastasis of OSCC. Additionally, a novel PDNS platform has been developed by optimizing non-covalent interactions between pure drugs to construct self-delivering immunostimulatory systems (Hao et al., 2024). The non-covalent interaction between chlorin e6 (Ce6) and NLG919 forms immunostimulatory systems that avoid toxicity and immunogenicity caused by excipients (Liu et al., 2024d). After intravenous injection, these systems passively accumulate in target tissues, enabling efficient PDNS and inducing immunogenic cell death, which in turn activates cytotoxic T lymphocytes (CTLs) and triggers immune responses.

Moreover, the delivered NLG919 inhibits indoleamine 2,3-dioxygenase 1 (IDO1), further enhancing the therapeutic efficacy. Another innovative class of PDNS employs ROS-responsive degradable linkers to conjugate DOX with polyethylene glycol (PEG), forming amphiphilic PEG-DOX conjugates (PEG-TK-DOX) that self-assemble into biologically activatable, ROS-responsive nanoparticles (Wang et al., 2024). This system efficiently encapsulates the hydrophobic PDNS agent pheophorbide A, enabling light-triggered chemo-photodynamic combination therapy and thus enhancing treatment efficacy.

Briefly, compared with classical photosensitive drugs, the aforementioned advancements suggest that PDNS has made significant strides recently, exhibiting strong selectivity and minimal side effects (An et al., 2024). Nonetheless, the application of PDNS still encounters several practical challenges, such as poor stability of photosensitizers *in vivo*, insufficient accumulation at target sites, and decreased efficacy caused by hypoxic environments (Gholami et al., 2024). To surmount these obstacles, researchers have devised various multidisciplinary drug approaches, which not only improve the stability and bioavailability of the core photosensitizers but also achieve targeted precision therapy and multifunctional applications, thereby bringing new hope for clinical treatment.

## 2 Advantages of PDNS

After exploring the specific composition and operational mechanism, it’s essential to highlight the numerous advantages these advanced therapeutic systems offer. These include the superior properties of nanocarriers, the ability to achieve synergistic multimodal therapy, the enhancement of photosensitizer efficacy, and the execution of targeted precision therapy. By examining these facets, a comprehensive understanding would be gained of how PDNS are revolutionizing the landscape of modern medicine.

### 2.1 Advantages of nanocarriers

The nanocarrier significantly enhances the biological distribution of photosensitizers and the enrichment of sites with translational efficacy varies. For instance, PEGylated liposomes improve circulation time but may trigger immune responses, whereas exosomes evade clearance mechanisms yet lack standardized production protocols (Dinakar et al., 2023; Lokesh et al., 2024). It shield them from degradation within the body, substantially prolong blood circulation time, and leverage the enhanced permeability and retention (EPR) effects to accumulate preferentially within target blood vessels (Kumar et al., 2024). The advantages of nanocarriers in the realm of medical therapy are well-documented and are becoming increasingly pivotal. Nanocarriers substantially enhance not only the biological distribution but also the enrichment of therapeutic photosensitizers at target sites (Dinakar et al., 2023).

**FIGURE 2:**
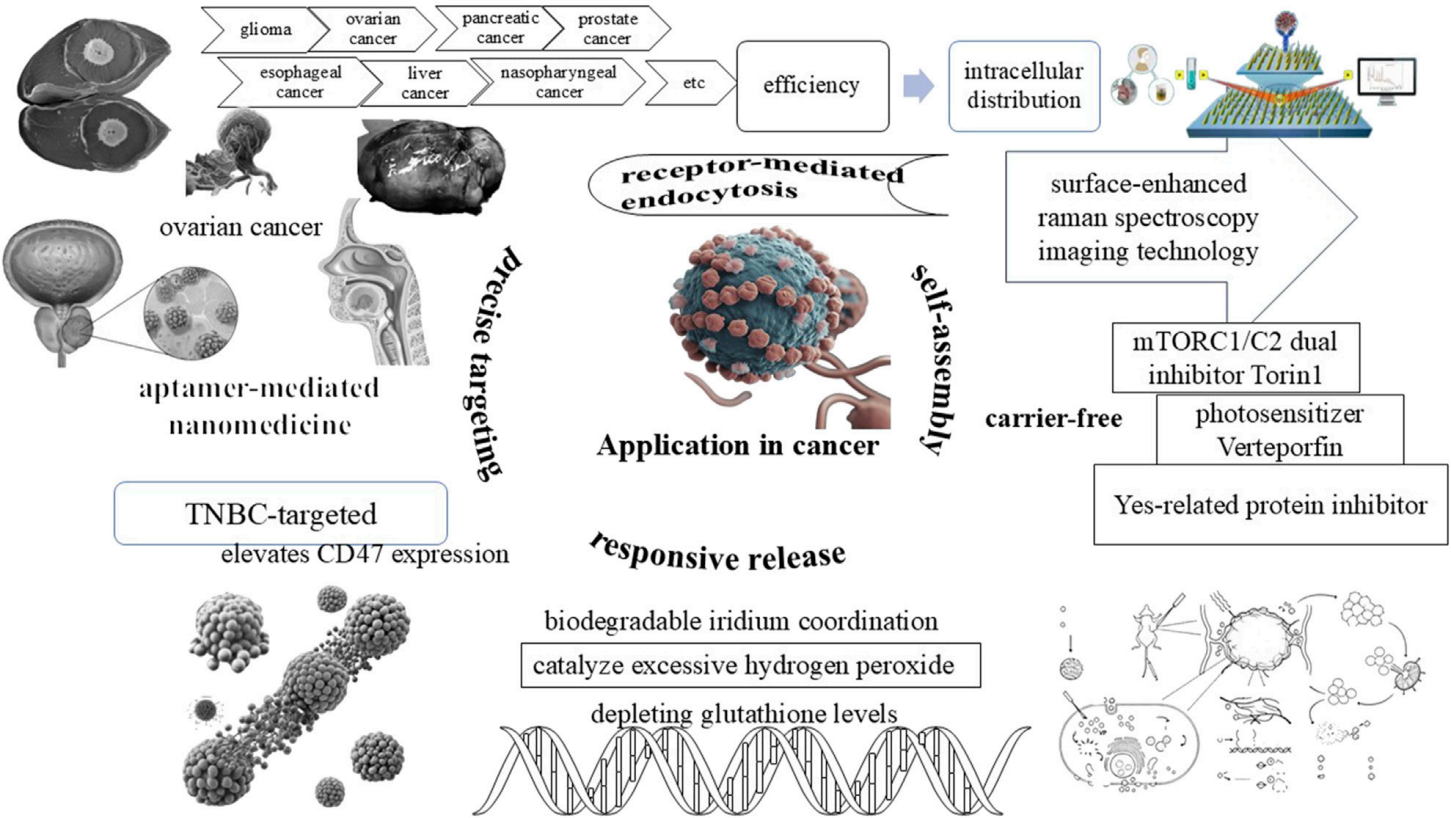
Advantages of nanocarriers in the PDNS design.

By encapsulating photosensitizers, these nanocarriers safeguard them from degradation within the body, thereby ensuring their stability and efficacy throughout the treatment process (D’Addio et al., 2012). Furthermore, the nanocarriers extend the blood circulation time of encapsulated photosensitizers, enabling a more sustained release and elevated bioavailability at the target site. This is partly accomplished through the EPR effects, which aid in the preferential accumulation within target blood vessels and tissues. Additionally, the nanocarriers can be specifically designed to target certain cell types, such as cancer stem cells, which often exhibit resistance to traditional therapies. By eradicating these tumor “seeds,” nanomedicine-based therapies have the potential to elicit more complete and durable responses (Li et al., 2023c). Moreover, the nanocarriers can facilitate the delivery of photosensitizers to deep tissues by inducing changes in tumor vasculature permeability (Figure 2).

Concisely, the advantages of nanocarriers in medical therapy are manifold, encompassing enhanced protection from degradation, prolonged blood circulation time, preferential accumulation at target sites, versatile design capabilities, and targeted delivery to specific cell types. These attributes position nanocarriers as a promising platform for the development of more efficacious and targeted therapies.

### 2.2 Achieving the synergistic effect of multimodal therapy

A versatile nanoplatform, endowed with a diverse array of functionalities such as photosensitizers, prodrugs, fluorescent dyes, and magnetic resonance contrast agents, represents a groundbreaking innovation that significantly amplifies the synergistic advantages of multimodal therapy. This remarkable advancement heralds a pivotal leap forward in the realm of medical treatment, promising unprecedented improvements in clinical outcomes. Researchers have meticulously designed a multifaceted nanoplatform that not only efficiently transports photosensitizers but also seamlessly integrates chemotherapy drugs or immunomodulators.

The innovative approach facilitates the powerful synergistic effect of multimodal therapy, marking a significant milestone in the evolution of medical treatment (Liu et al., 2024c). This strategy holds immense promise for addressing a multitude of diseases, notably cancer and bacterial infections. In the realm of cancer immunotherapy, a prodrug has been engineered to respond to the tumor microenvironment, inducing pyroptosis and thereby activating the immune system for multimodal synergistic therapy. By harnessing the heat generated, the efficacy of co-administered therapeutic agents is augmented, stimulating a more robust attack on cancer cells. It has demonstrated that the synergy between the prodrug and the immune system leads to improved therapeutic outcomes (Xiao et al., 2021).

Analogously, in the fight against bacterial infections, the synergistic effect of combination therapy on ovarian cancer cells was investigated. Results indicated that the combination could significantly enhance cytotoxicity, underscoring the potential of multimodal therapy in treating this aggressive disease. Furthermore, the integration was found to effectively eradicate target cells, highlighting the importance of combining diverse therapeutic modalities to achieve optimal treatment results (Wang et al., 2022). More recently, a porous graphitic nanosheet (PGNS) synthesized via the metal-organic MOF method was introduced to bolster the synergistic therapeutic effect, including ferroptosis and starvation therapy. By reversing the hypoxic tumor microenvironment, the efficacy of these therapies is enhanced, demonstrating the potential of MOFs in multimodal cancer treatment.

In the broader context, the development of such versatile nanoplatforms signifies a transformative shift in the landscape of therapeutic interventions. By harnessing the unique properties of nanotechnology, researchers are able to create highly targeted and effective treatment strategies that can address complex medical challenges with greater precision and efficacy. As research continues to evolve, the potential applications of these nanoplatforms are likely to expand, further revolutionizing the field of medicine and improving patient outcomes.

### 2.3 Improving the efficacy of photosensitizers

The effectiveness of clinical treatment is profoundly impacted by the stability and bioaccumulation of core photosensitizers within the body. This is attributed to its effective shielding from *in vivo* degradation, prolonged circulation time in the bloodstream, and preferential accumulation in targeted blood vessels, facilitated by the enhanced permeability and retention effects. These PDNS can be designed with pH or redox sensitivity, enabling the release of encapsulated photosensitizers in response to specific target microenvironments. This approach not only enhances local concentration but also minimizes damage to normal tissues.

Stability is a crucial factor in this context. Amphiphilic phospholipid-based riboflavin derivatives have been developed for tumor-targeting PDNS, exhibiting improved stability and bioavailability. Enhancing stability is essential for maintaining the photosensitizing capacity of these molecules over extended periods, ensuring sustained therapeutic activity (Beztsinna et al., 2016). In parallel, nano-PROTACs have been utilized to degrade specific proteins, such as BRD4, amplifying DNA damage and underscoring the importance of molecular engineering in augmenting the therapeutic potential of photosensitizers, although this does not directly address photosensitizer stability. The concentration of photosensitizers directly correlates with their therapeutic effect (Zhao et al., 2023). Bioaccumulation is another pivotal factor influencing the effectiveness of PDNS. Enhancing the bioaccumulation of photosensitizers can be achieved through various strategies, including chemical modification, the application of nanotechnology, biomolecular templating, optimization of drug delivery routes, and the adjustment of physical and chemical properties. These approaches serve to elevate their concentration and efficacy within biological organisms, thereby fortifying their application potential. However, it is critical to balance such enhancements with the potential toxicities associated with increased concentrations. Separate studies have provided valuable insights into the complex interactions between nanomaterials and biological systems (Haghighat et al., 2021). These insights can inform strategies for optimizing photosensitizer concentrations *in vivo*, ensuring maximum therapeutic effect while minimizing toxicity (Resende et al., 2025).

Advanced analytical techniques, such as asymmetrical flow field-flow fractionation with on-line detection, enable the investigation of drug retention within liposomal PDNS and drug transfer kinetics. This understanding is essential for designing nanocarriers that can stabilize and effectively deliver photosensitizers to target sites (Hinna et al., 2016). Recently, a self-monitoring and self-delivery system for photosensitizer-doped PDNS has been introduced, achieving highly effective combination cancer therapy both *in vitro* and *in vivo*. This system demonstrates the potential of integrating advanced technologies to enhance the efficacy and safety of PDNS (Zhang et al., 2015).

### 2.4 Targeted precision therapy

Targeted precision therapy has emerged as a cornerstone in the advantage of PDNS, leveraging cutting-edge surface modification techniques, photo-control mechanisms, and seamless integration with imaging technologies. These advanced PDNS exhibit remarkable precision in targeting, substantially elevated therapeutic efficacy, and a significant reduction in collateral damage. It herald a new era of more effective and personalized cancer therapies within the precision medicine paradigm.

Firstly, surface modification techniques empower the specific recognition of cell surface markers, thereby facilitating highly precise treatment. Various strategies have been employed for the surface modification of PDNS, primarily involving the incorporation of molecules such as antibodies, peptides, and aptamers. It has exhibited efficacy in achieving substantially higher concentrations at target sites. For example, antibody-conjugated PDNS (e.g., HER2-targeted systems) show promise, clinical trials reveal limitations such as antigen heterogeneity and off-target binding in non-cancer tissues (Yan et al., 2024). Similarly, peptide-modified nanocarriers based on αvβ3 integrin have been successfully utilized in the precise treatment of melanoma (Hapuarachchige et al., 2014).

Furthermore, research has progressed to develop photo-controlled targeted PDNS. These innovative PDNS undergo conformational changes under light irradiation, enabling the selective release of photosensitizers and the precise elimination of target cells. This enhancement not only bolsters the safety profile of the treatment but also significantly augments its effectiveness. Recently, studies have reported the development of two-photon excited peptide PDNS for precise therapy. These PDNS leverage the unique properties of two-photon excitation to achieve deeper tissue penetration and further minimize collateral damage (Cao et al., 2021). Additionally, smart peptide-based supramolecular photodynamic metallo-PDNS, designed through multicomponent coordination self-assembly, have shown promising results in targeted therapy (Li et al., 2018). These PDNS offer enhanced stability and controlled release of photosensitizers, further refining therapeutic precision.

Moreover, advancements in imaging technologies have facilitated the development of PDNS that integrate NIR-II fluorescence imaging with oxygen self-sufficiency for precise enhanced photodynamic therapy. These platforms enable real-time monitoring of photosensitizer distribution and oxygen levels within tumors, thereby guiding the administration of photodynamic therapy for optimized therapeutic outcomes (Li et al., 2022b). Metal-free PDNS, consisting of water-soluble photosensitizers and adenosine triphosphate, have also been explored for efficient and precise therapy. These assemblies present a biocompatible and cost-effective alternative to metal-based PDNS, while maintaining high therapeutic efficacy (Li et al., 2021).

To sum up, the integration of advanced therapeutic systems of PDNS highlights numerous advantages, including the superior properties of nanocarriers that enhance the biological distribution and site enrichment of photosensitizers. Encapsulating these photosensitizers within nanocarriers protects it from degradation *in vivo*, prolongs its blood circulation time, and leverages enhanced permeability and retention effects to accumulate preferentially in target blood vessels. Furthermore, versatile nanoplatform, incorporating a diverse range of functionalities such as photosensitizers, prodrugs, fluorescent dyes, and magnetic resonance contrast agents, revolutionizes multimodal therapy by amplifying its synergistic benefits. Additionally, the stability and bioaccumulation of core photosensitizers within the body are crucial for clinical treatment effectiveness, attributed to their effective shielding from degradation, prolonged circulation, and targeted accumulation facilitated by enhanced permeability and retention. Finally, targeted precision therapy has become a cornerstone, utilizing cutting-edge surface modification techniques, photo-control mechanisms, and seamless integration with imaging technologies to maximize the advantages of PDNS.

## 3 Application of PDNS

### 3.1 Application mechanisms in cancer treatment

**FIGURE 3:**
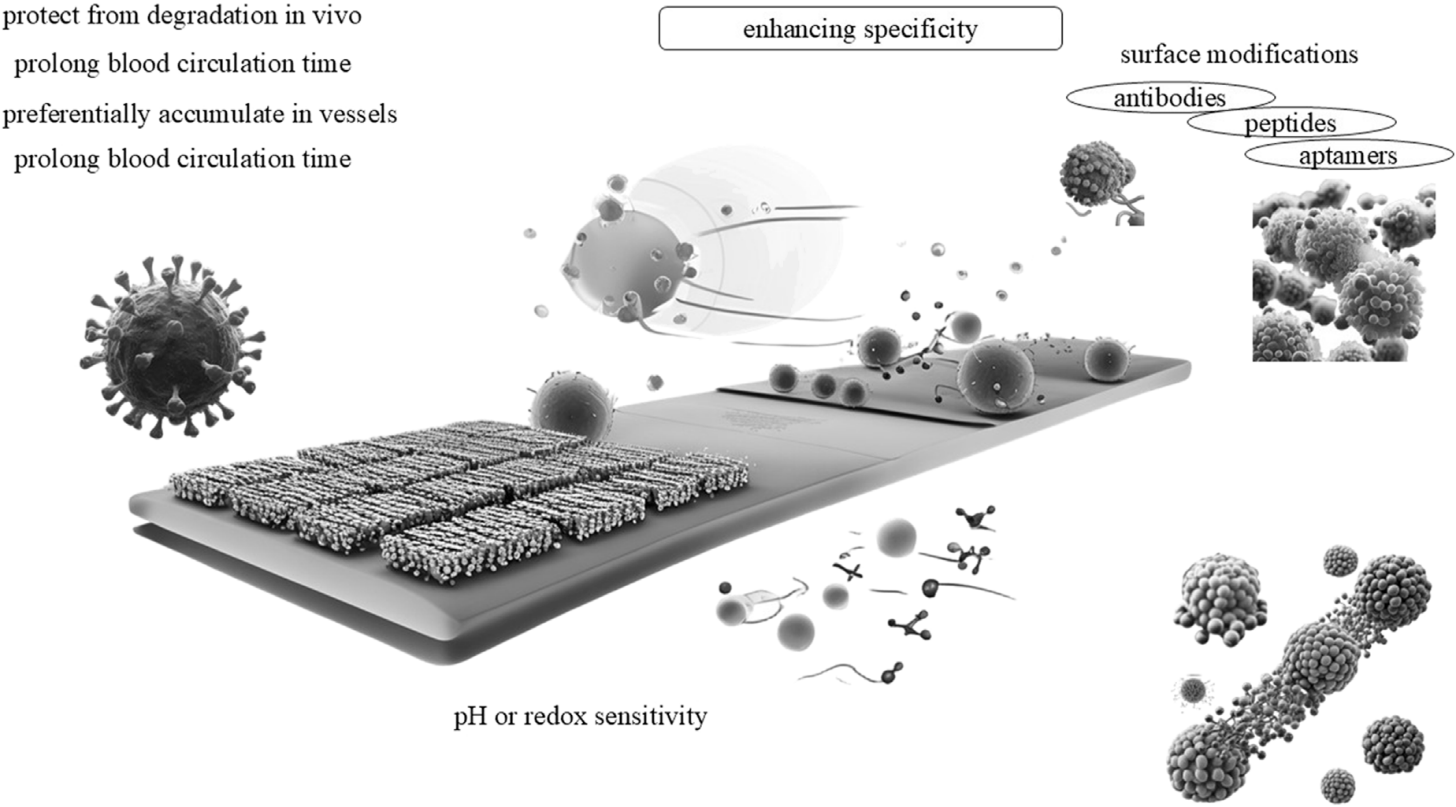
Application mechanisms in cancer treatment for PDNS.

The application of PDNS in cancer treatment has manifested remarkable progress, particularly through mechanisms like receptor-mediated endocytosis, self-assembly, responsive release, and precise targeting (Figure 3). Utilizing chemical modifications on its nanocarriers such as gold nanorods (GNRs), PDNS can selectively bind to receptors on the surface of tumor cells. This targeted binding facilitates the entry of PDNS into cancer cells via this mechanisms, significantly enhancing its efficiency and intracellular distribution (Tang et al., 2021). In practical applications, the integration of surface-enhanced raman spectroscopy (SERS) imaging technology provides a powerful method for the real-time visualization of the location and distribution of photosensitizers. This guarantees precise drug delivery to the target area, further optimizing treatment outcomes and minimizing off-target effects.

In addition, self-assembly strategy is of great significance in the construction of efficient anti-cancer platforms. Through this technology, various therapeutic molecules can form stable nanostructures, which improve its drug stability, biocompatibility, and achieve synergistic therapeutic effects. For instance, a carrier-free nanomedicine formulation consisting of mTORC1/C2 dual inhibitor Torin1, photosensitizer Verteporfin, and Yes-related protein inhibitor has been developed (Liu et al., 2024a). This formulation not only enhances drug efficacy but also induces apoptotic cell death, anti-angiogenesis, and immunogenic cell death effects. Another example is the formation of non-metallic helical nanofibers through the co-assembly of cationic porphyrins and ATP, which exhibits potential due to its ease of availability, compatibility, simple preparation, and robust performance (Li et al., 2021). Through systemic blood circulation, the agents can enhance the photosensitizer delivery ability at tumor sites, achieving efficient cancer treatment.

Moreover, responsive release mechanisms are also crucial. A typical example is acidity responsive biodegradable iridium coordination (IPC) nanomedicine (Li et al., 2025). This drug has the ability to catalyze excessive hydrogen peroxide into oxygen within the tumor microenvironment, concurrently depleting glutathione levels in cancer cells. This dual action bolsters the effectiveness of PDNS and immunotherapy. Furthermore, the IPC nanomedicine can ameliorate tumor hypoxia, inducing more immunogenic cell death by intensifying the PDNS response, and thereby achieving synergistic inhibition of tumor growth. An additional example is GSH-responsive prodrug-based nanomedicine, which encapsulates a mitomycin C (MMC) prodrug and the photosensitizer Chlorine6 (Ce6) within its structure (Yu et al., 2022), accompanied by surface modifications with tumor-specific aptamers. Upon reaching the tumor site, the GSH response mechanism initiates drug release while simultaneously consuming glutathione within the tumor, resulting in a severe redox imbalance and augmenting the efficacy of Ce6-based photodynamic therapy (Shi et al., 2024).

Ultimately, realizing efficient and safe cancer treatment with precise targeting holds the key to achieving high efficacy and low toxicity. A quintessential example lies in aptamer-mediated nanomedicine. By selectively binding to specific receptors on the surface of tumor cells via aptamers, the PDNS can accomplish precise recognition and targeting of tumor cells. This strategy not only enhances the accumulation at the tumor site but also circumvents damage to normal tissues (Yan et al., 2024). Another illustrative example is TNBC-targeted nanomedicine (CPU) possessing self-promoting targeting properties. This drug elevates CD47 expression through chemotherapy, thereby accomplishing synergistic chemotherapy/photodynamic therapy. The experimental results demonstrate that CPU exhibits superior tumor targeting ability both *in vitro* and *in vivo* with dose-limiting hepatotoxicity, which can substantially amplify the effects of chemotherapy and photodynamic therapy while mitigating significant adverse reactions (Liu et al., 2024b).

### 3.2 Application in various non-oncological diseases

In addition to its extensive and well-established applications in the field of cancer treatment, the PDNS have also exhibited considerable promise in treating a diverse array of non-oncological diseases, including acne, psoriasis, dry eye disease, and cardiovascular disorders. Within dermatological applications, PDNS have demonstrated significant potential in the treatment of acne and psoriasis. Specifically, in the treatment of acne, a biocompatible nano-formulation that integrates methylene blue and salicylic acid has been found to effectively reduce acne lesions and decrease sebum production (Lee et al., 2023). Regarding psoriasis, Polydopamine/IR820 nanoparticles have emerged as a promising topical phototheranostic agent, capable of inhibiting psoriasiform lesions through a dual-modality approach combining photothermal and photodynamic therapies. These image-guided phototheranostic nanoparticles have exhibited significant potential for non-invasive topical administration in the treatment of psoriasis (Nirmal et al., 2022).

Similarly, in ophthalmic treatments, especially for fundus diseases like retinoblastoma, PDNS present broad and promising application prospects. To address this condition, researchers have devised a novel photodynamic nano-platform that integrates liposomes with indocyanine green (ICG), addressing several inherent challenges associated with ICG, such as its susceptibility to quenching, self-aggregation, and instability. This innovative approach not only encapsulates ICG within liposomes to prevent its clearance through systemic circulation, thereby ensuring its stability within the body, but also facilitates imaging-guided photothermal therapy (Liu et al., 2022). Furthermore, another example known as FA-DOX-ICG-PFP@Lip, has also been devised. This folate-receptor (FR)-targeted, laser-activatable liposome carries doxorubicin (DOX), ICG, and liquid perfluoropentane (PFP). It is specifically designed for photoacoustic/ultrasound dual-modal imaging-guided chemo/photothermal therapy of retinoblastoma. These multifunctional PDNS-based formulations exhibit superior tumor targeting ability and serve as highly effective dual-modality contrast agents, both *in vivo* and *in vitro* (Li et al., 2022a).

In the realm of cardiovascular diseases, PDNS also represent a promising therapeutic approach. Various photodynamic nanoparticulate delivery systems for berberine have been developed, including lipid-based, inorganic-based, and polymeric-based nanoparticles. These berberine nanocarriers significantly amplify its therapeutic efficacy and capabilities by prolonging its half-life, enhancing solubility, and facilitating superior permeation and targeted delivery. The employment of berberine-mediated photodynamic therapy via photodynamic nanotechnology stands as a highly encouraging strategy for addressing cardiovascular diseases (Marques et al., 2023). Another notable illustration is the formulation of Ce6-loaded liposomes prepared through the film dispersion technique, subsequently conjugated with CD68 antibody on the liposomal surface via a covalent crosslinking reaction, thereby yielding CD68-modified Ce6-loaded liposomes. It exhibits inhibitory effects on MOVAS migration and promote cholesterol efflux in foam cells. Consequently, the Ce6-loaded liposomes represent promising nanocarriers for photodynamic therapy in the context of atherosclerosis. Additionally, PDNS also have shown promising potential in combating bacterial infections. Antibiotic micelles combined with PDT were developed, where antibacterial efficacy was enhanced through ROS generation (Yang et al., 2023). A glucose-responsive nanodrug system for diabetic wounds was designed, in which endogenous glucose was utilized to improve ROS-mediated killing of resistant bacteria (Zhang et al., 2024). In both approaches, PDNS are employed to address infections, particularly in cases involving antibiotic resistance and chronic wounds. Concisely, beyond its well-established application in cancer treatment, PDNS have shown great promise in treating various non-oncological diseases. The incorporation of photodynamic nanotechnology of PDNS enables more precise and targeted drug delivery, enhancing therapeutic efficacy across these disease areas and highlighting the transformative potential in medical therapy (Zou et al., 2023).

### 3.3 Combined application with immunotherapy

PDNS have emerged as potent allies in the field of cancer immunotherapy, exhibiting several notable advantages. By harnessing the immune system’s innate ability to identify, attack, and eliminate cancer cells, immunotherapy holds great promise. However, traditional immunotherapy faces challenges such as limited response rates and adverse effects, impeding its broader application (Yu et al., 2023). PDNS, with its immunostimulatory properties, have the potential to overcome these limitations and revolutionize cancer treatment (Guo et al., 2022).

One innovative approach involves the use of integrated photodynamic nanoplatform, termed HN-HFPA, which has demonstrated positive efficacy in boosting the anti-tumor immunity of TNBC. This integrated platform reeducates tumor-associated macrophages by interfering with arginine metabolism. Specifically, L-arginine loaded within the nanoplatform’s hollow cavity is utilized to produce nitric oxide, which is crucial for tumor therapy and the induction of immunogenic cell death. Simultaneously, the surface of the nanoplatform is coated with L-norvaline-modified hyaluronic acid, which suppresses Arg-1 of M2 TAMs, skewing the arginine metabolism balance, reversing TAM polarization, and amplifying the antitumor immune response (Chen et al., 2024).

Furthermore, a pH/enzyme dual-sensitive polymeric micelle PDNS has been designed for synergistic tumor immuno-photodynamic therapy. It incorporates a photosensitizer and PD-L1 antibody, and is equipped with a sheddable PEG coating for codelivery. In the tumor microenvironment, it sequentially triggers PEG shedding and PD-L1 antibody release, facilitating local immune checkpoint blockade (ICB). Light irradiation enhances tumor cell immunogenic death, promotes CD8^+^ T cell recruitment, and reduces immune-related adverse events (IRAEs).

Another example is the PolyMN-TO-8, constructed through self-assembly of MTX-MPEG2000, NPX-2S, and TO-8. It exhibits excellent stability and responsiveness to carboxylesterase and glutathione. Upon laser triggering, it induces pyroptosis-mediated immunogenic cell death, enhancing dendritic cell maturation and accelerating CD8^+^ T cell infiltration into tumors. This eliminates immune escape and achieves a high tumor inhibition rate, further underscoring its potential as a powerful therapeutic agent in cancer immunotherapy (Wang et al., 2023b).

In short, PDNS offer a promising new approach when combined with immunotherapy. Its advanced design and unique properties enable precise, noninvasive tumor therapy with enhanced therapeutic effects and reduced side effects. This innovative strategy highlights the potential for developing next-generation smart nanomedicines that can significantly improve cancer treatment outcomes.

### 3.4 Application in personalized medicine

Personalized medicine, a revolutionary and rapidly advancing field in modern healthcare, lies at the heart of tailoring medical treatments to the unique characteristics of each patient. It takes into account a multitude of factors such as genomic sequences, disease type and stage, and physiological function to develop customized treatment plans that are more effective and have fewer side effects. PDNS have emerged as a key component within the realm of personalized medicine, garnering significant attention due to its exceptional potential in enabling highly personalized therapy. By precisely designing and optimizing these PDNS to target specific disease types, genetic markers, and physiological conditions, it align seamlessly with the principles of personalized medicine. This approach not only maximizes treatment efficacy but also minimizes side effects and enhances the overall safety, all of which are crucial aspects in personalized medicine.

A novel NIR-activated hybrid nanodrug, UCNPs-F127@Cur, has been developed, leveraging upconversion nanoparticles, Pluronic F127, and curcumin. It effectively promotes GSC apoptosis, enhances intracellular reactive oxygen species production, and inhibits GSC pluripotency gene expression, suppressing tumor growth *in vivo* under 980 nm light. Transcriptome analysis reveals its mechanism involves cell cycle arrest and GSC differentiation by blocking Wnt-β-catenin and Jak-Stat pathways. Despite promising preclinical results (e.g., UCNPs-F127@Cur), clinical translation is hindered by batch variability in nanodrug synthesis and limited patient stratification tools (Jing et al., 2024).

**FIGURE 4:**
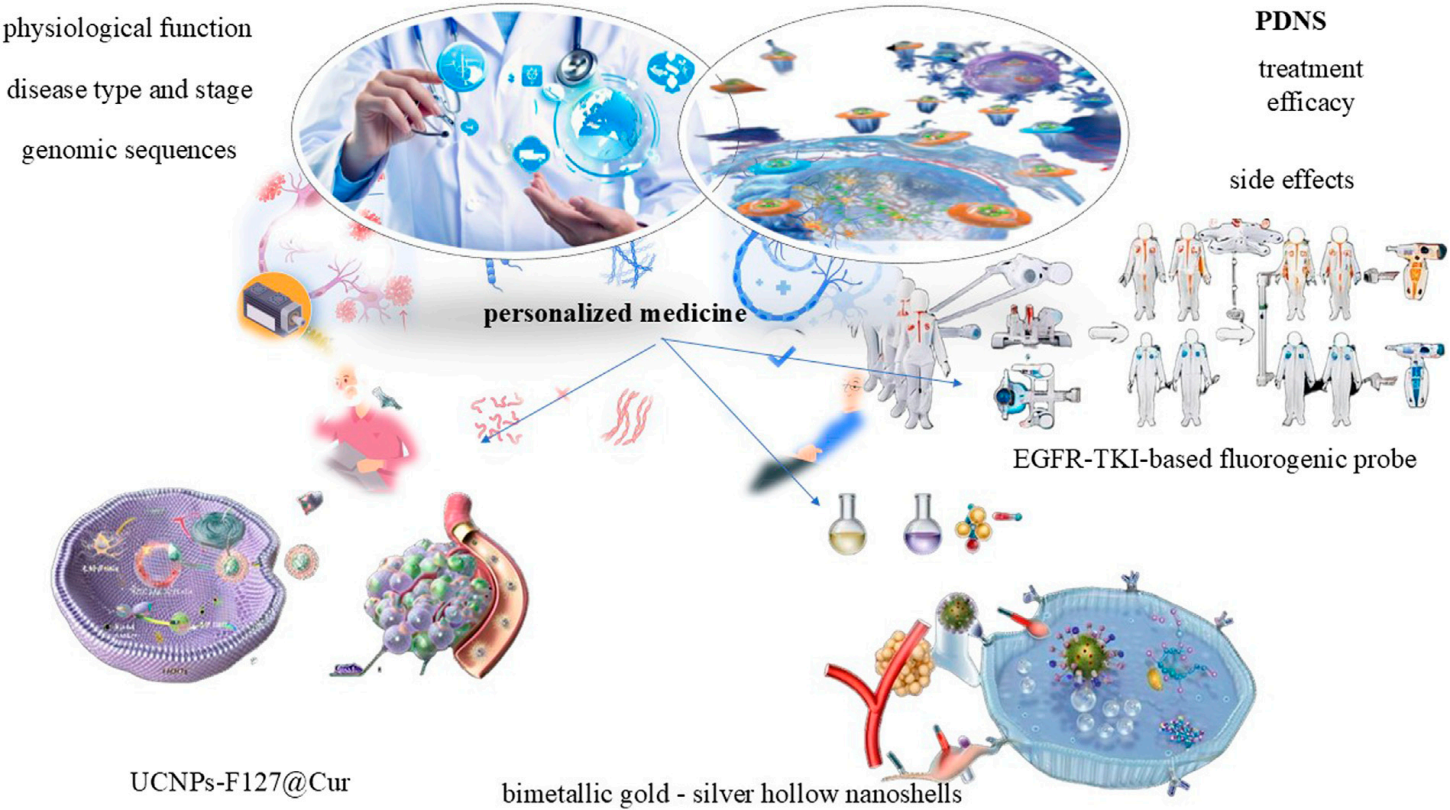
Application in personalized medicine for PDNS.

An example integrates bimetallic gold - silver hollow nanoshells with remarkable NIR absorption, the tyrosine kinase inhibitor pyrotinib, and herceptin. It embodies a personalized medicine strategy by providing multiple targeted therapeutic effects, such as chemotherapy, photothermal therapy, oxidative stress induction, and immune response activation. This multimodal treatment also boosts the upregulation of genes in TNF and NF - κB signaling pathways, enhancing immune activation and immunogenic cell death. *In vivo* experiments verify a significant shrinkage of tumor volume after treatment, proving the potential effectiveness and personalized treatment value of these nanocarriers (Zhao et al., 2024b). When combined with computed tomography imaging, a strong correlation is observed between EGFR-TKI therapy response and probe labeling. These findings demonstrate that HX103-based FACS provides highly predictive performance for EGFR-TKI response. It offering the potential to stratify NSCLC patients for EGFR-TKI treatment based on individual genetic profiles. The EGFR-TKI-based probe, HX103, therefore, holds promise for use in precision medicine trials, aiming to tailor treatments to patient-specific needs (Deng et al., 2022). It is suggested that novel NIR-activated PDNS and multicolor FACS techniques utilizing specific probes show promise in personalized medicine for GBM and NSCLC treatment, tailored to individual patients’ genetic profiles (Figure 4).

Despite the promising efficacy demonstrated by PDNS, several clinical limitations are encountered. First, the tissue penetration of light is restricted, which limits treatment depth, where near-infrared activatable agents were suggested to improve efficacy (Cheah et al., 2018). Second, challenges related to stability and controlled drug release *in vivo* are faced by carrier-free nanodrugs (Lin et al., 2024). Additionally, off-target effects and complex synthesis issues are associated with dual-targeting nanomaterials (Zhang et al., 2023). Potential systemic toxicity is introduced when immunotherapy is integrated (Liu et al., 2024a), while the difficulty in achieving synergistic effects without exacerbating side effects was underscored. These issues collectively hinder clinical translation, necessitating further optimization of PDNS design and delivery strategies.

## 4 Current challenges and future prospects

As an emerging treatment method that combines the advantages of photodynamic therapy and nanotechnology, PDNS have demonstrated significant advantages in enhancing therapeutic outcomes while reducing systemic toxicity; however, the translational efficacy of these systems varies widely depending on the choice of photosensitizer and nanocarrier. For instance, while liposomal nanocarriers improve drug stability and targeting via the EPR effect, their rapid clearance by the reticuloendothelial system limits their clinical utility compared to exosome-based systems, which exhibit superior biocompatibility and prolonged circulation. Similarly, porphyrin-based photosensitizers dominate clinical use due to their strong ROS generation, yet their poor tissue penetration contrasts with emerging near-infrared (NIR) agents like indocyanine green (ICG), which enable deeper tumor targeting. These trade-offs highlight critical gaps in optimizing PDNS for specific clinical scenarios. So, there are still some challenges in the efficient design, and precise delivery and release of nanocarriers. This section will analyze these challenges and explore possible future research and development strategies.

### 4.1 Design of efficient photosensitizer

The design and development of efficient photosensitizers harbor immense potential in clinical applications. To circumvent the limitations of classical photosensitizers, researchers are actively pursuing innovative approaches that fuse nanotechnology, material science, and herbal medicine (Bilia et al., 2025). This interdisciplinary strategy aims to surmount challenges associated with tissue penetration depth, light utilization efficiency, and biocompatibility, ultimately enhancing both efficacy and safety of clinical application.

Among which advancements in nanotechnology and material science have catalyzed the exploration of novel photosensitizers. Formulations under investigation include carbon dots, upconversion nanoparticles, plasmonic nanomaterials, organic small molecules exhibiting near-infrared absorption properties, and MOFs. These novel photosensitizers offer numerous advantages, such as broadened absorption spectra, enhanced photostability, and facilitated deep tissue penetration. By fine-tuning particle size and surface modification, researchers can customize these photosensitizers to meet specific therapeutic needs. Furthermore, the incorporation of herbal medicine into photosensitizer design presents a promising avenue (Zhao et al., 2024c). Many herbal compounds exhibit significant potentials due to their superior biocompatibility, reduced toxicity, and multi-target synergies. Key compounds such as curcumin, hypericin, and berberine demonstrate intrinsic photodynamic activity, with structural modifications (e.g., glycosylation of anthraquinones) further enhancing efficacy (Sulaiman et al., 2022). Their alignment with traditional medicine frameworks may facilitate regulatory approval, given their established safety profiles. Additionally, these herbal compounds possess near-infrared absorption properties, making them suitable for deep-tissue applications and wound healing (Zhao et al., 2024a). Investigative efforts should be directed toward the rational design of herbal-derived photosensitizers, including glycosylated hypericin derivatives that demonstrate enhanced penetration in preclinical models, as well as curcumin analogs with improved bioavailability, to capitalize on their multi-target synergies and regulatory advantages, while concurrently optimizing formulations, refining light activation protocols, and expanding clinical validation to maximize their therapeutic and industrial applications.

Essentially, the design of efficient photosensitizers constitutes a pivotal area of research. By embracing an interdisciplinary approach that amalgamates nanotechnology, material science, and herbal medicine, researchers can overcome current limitations and develop next-generation photosensitizers with superior properties. Future research endeavors should concentrate on validating these novel photosensitizers in preclinical models. Future strategies should focus on molecular engineering to enhance ROS quantum yields. For example, covalent conjugation of IR780 with cyclodextrin improves solubility without compromising photostability. Additionally, TCM-derived photosensitizers show synergistic effects (Liang et al., 2022) but require rigorous pharmacokinetic validation (Sun et al., 2025).

### 4.2 Precise delivery and release of nanocarriers

The precise delivery and release of nanocarriers also play a key role, as it not only safeguards its photosensitizers from degradation but also facilitates targeted drug delivery (Liang et al., 2025). However, some existing nanocarriers exhibit poor stability *in vivo* and are readily captured by the reticuloendothelial system, resulting in ineffective drug delivery. Additionally, the release mechanism of these nanocarriers is relatively limited, hindering on-demand release and subsequently impacting the therapeutic effect and/or toxicity. To address these issues, researchers are exploring intelligent precise delivery and release of nanocarriers, such as pH-sensitive, redox-sensitive, or enzyme-sensitive nanomaterials. Beyond pH/redox sensitivity, dual-responsive systems (e.g., MMP-9/enzyme-triggered release) and surface topology optimization can enhance tumor-specific accumulation. Clinical adoption requires addressing scalability of stimuli-responsive materials and validating safety in large-animal models. Recently, multifunctional nanocarriers have been devised to overcome these limitations by integrating numerous stimuli-responsive mechanisms into a singular platform. These systems can respond to the characteristics including an acidic extracellular pH, elevated GSH levels, and overexpressed specific enzymes, thereby augmenting the specificity and efficiency. A dual-responsive nanocarriers that combine pH sensitivity with redox responsiveness have demonstrated potential in overcoming the hurdles associated with intracellular delivery.

Furthermore, researchers are investigating the incorporation of external stimuli-responsive factors, such as NIR light or ultrasound, into nanocarrier designs. These external triggers provide spatiotemporal control, enabling precise targeting, even in deep tissues. The combination of internal and external stimuli responsiveness further enhances the adaptability of precise delivery and release of nanocarriers to complex physiological conditions. Additionally, surface modifications with targeting ligands, such as antibodies, peptides, or aptamers, can enhance the selectivity, reducing off-target effects and improving safety profiles. Future research should concentrate on developing robust precise delivery and release of nanocarriers platforms that integrate multiple functions while maintaining high therapeutic efficacy and low toxicity. Moreover, comprehensive *in vivo* evaluations and translational studies are also imperative to bridge the gap between laboratory research and clinical applications.

## 5 Conclusion

PDNS, as an emerging therapeutic approach, has made significant advancements in both basic research and clinical applications in recent years. This class of drugs consists of photosensitizers and nanocarriers, each playing a crucial role. PDNS have emerged as a transformative therapeutic modality, elegantly merging photosensitizers with nanocarriers to address traditional challenges. This integration not only bolsters phototherapeutic efficacy but also introduces remarkable advantages in targeted precision therapy for a broad spectrum of diseases, encompassing both oncological and non-oncological conditions. The present manuscript delves into the composition, mechanisms of action, advantages, applications, and future perspectives of PDNS. It underscores how PDNS seamlessly integrate photosensitizers with its corresponding nanocarriers to elevate phototherapeutic efficacy, achieve precise targeting of diseased cells, minimize damage to healthy tissues, and transcend limitations associated with poor stability and restricted tissue penetration. Furthermore, the manuscript explores the advantages of PDNS in cancer treatment through mechanisms like receptor-mediated endocytosis, self-assembly, responsive release, and precise targeting, its application in non-oncological disorders including acne, psoriasis, dry eye disease, and cardiovascular disorders, its synergistic integration with immunotherapy, and its pivotal role in personalized medicine due to its exceptional potential in enabling highly personalized therapy.

It suggests that the PDNS represent a groundbreaking advancement in therapeutic technology, merging the capabilities of photosensitizers and nanocarriers. The strong selectivity and minimal side effects make it ideal for targeted precision therapy across various diseases, including cancer and non-oncological conditions like acne and cardiovascular diseases. The nanocarriers have revolutionized cancer treatment. It overcome classical photosensitizers’ limitations, enhancing efficacy and safety via targeted delivery and synergistic therapy, thus offering hope for personalized clinics. By harnessing the immune system’s innate ability to identify, attack, and eliminate cancer cells, immunotherapy holds great promise. PDNS have emerged as a component within the realm of personalized medicine, garnering significant attention due to its potential in enabling highly personalized therapy. By precisely designing and optimizing these PDNS to target specific disease types, genetic markers, and physiological conditions, it align seamlessly with the principles of personalized medicine. However, despite the above promising developments, challenges persist in designing more efficient photosensitizers, and ensuring precise delivery and release of nanocarriers. Addressing these issues will be crucial for fully realizing the transformative potential of PDNS in future.

## Data Availability

The original contributions presented in the study are included in the article/supplementary material, further inquiries can be directed to the corresponding author.

## References

[B1] An G. Zheng H. Guo L. Huang J. Yang C. Bai Z. (2024). A metal-organic framework (MOF) built on surface-modified Cu nanoparticles eliminates tumors via multiple cascading synergistic therapeutic effects. J. Colloid Interface Sci. 662, 298–312. 10.1016/j.jcis.2024.02.055 38354557

[B2] Beztsinna N. Tsvetkova Y. Bartneck M. Lammers T. Kiessling F. Bestel I. (2016). Amphiphilic phospholipid-based riboflavin derivatives for tumor targeting nanomedicines. Bioconjug Chem. 27 (9), 2048–2061. 10.1021/acs.bioconjchem.6b00317 27412680

[B3] Bilia A. R. Ballerini R. Qu L. Wang M. (2025). Traditional Chinese herbal medicine in European Union: state of art, challenges, and future perspectives focusing on Italian market. Chin. Herb. Med. (CHM) 17 (1), 3–18. 10.1016/j.chmed.2024.11.008 39949801 PMC11814246

[B4] Cao H. Qi Y. Gao X. Wei Z. J. Xia J. Wang L. (2021). Two-photon excited peptide nanodrugs for precise photodynamic therapy. Chem. Commun. (Camb) 57 (18), 2245–2248. 10.1039/d0cc08219h 33554229

[B5] Chang R. Zhao L. Y. Xing R. R. Li J. B. Yan X. H. (2023). Functional chromopeptide nanoarchitectonics: molecular design, self-assembly and biological applications. Chem. Soc. Rev. 52 (8), 2688–2712. 10.1039/d2cs00675h 36987746

[B6] Cheah H. Y. Gallon E. Dumoulin F. Hoe S. Z. Japundžić-Žigon N. Glumac S. (2018). Near-infrared activatable phthalocyanine-poly-L-glutamic acid conjugate: enhanced *in vivo* safety and antitumor efficacy toward an effective photodynamic cancer therapy. Mol. Pharm. 15 (7), 2594–2605. 10.1021/acs.molpharmaceut.8b00132 29763568

[B7] Chen Y. Shu X. Guo J. Y. Xiang Y. Liang S. Y. Lai J. M. (2024). Nanodrugs mediate TAMs-related arginine metabolism interference to boost photodynamic immunotherapy. J. Control Release 367, 248–264. 10.1016/j.jconrel.2024.01.045 38272398

[B8] D'Addio S. M. Saad W. Ansell S. M. Squiers J. J. Adamson D. H. Herrera-Alonso M. (2012). Effects of block copolymer properties on nanocarrier protection from *in vivo* clearance. J. Control Release 162 (1), 208–217. 10.1016/j.jconrel.2012.06.020 22732478 PMC3416956

[B9] Deng H. Lei Q. Wang C. Wang Z. Chen H. Wang G. (2022). A fluorogenic probe for predicting treatment response in non-small cell lung cancer with EGFR-activating mutations. Nat. Commun. 13 (1), 6944. 10.1038/s41467-022-34627-5 36376325 PMC9663578

[B10] Dinakar Y. H. Rajana N. Kumari N. U. Jain V. Mehra N. K. (2023). Recent advances of multifunctional PLGA nanocarriers in the management of triple-negative breast cancer. AAPS PharmSciTech 24 (8), 258. 10.1208/s12249-023-02712-7 38097825

[B11] Ding F. Liu J. Ai K. Xu C. Mao X. Liu Z. (2024). Simultaneous activation of pyroptosis and cGAS-STING pathway with epigenetic/photodynamic nanotheranostic for enhanced tumor photoimmunotherapy. Adv. Mater 36 (7), e2306419. 10.1002/adma.202306419 37796042

[B12] German N. Popov A. Ramanaviciene A. (2024). Reagentless glucose biosensor based on combination of platinum nanostructures and polypyrrole layer. Biosens. (Basel) 14 (3), 134. 10.3390/bios14030134 PMC10968463 38534241

[B13] Gholami A. Mohkam M. Soleimanian S. Sadraeian M. Lauto A. (2024). Bacterial nanotechnology as a paradigm in targeted cancer therapeutic delivery and immunotherapy. Microsyst. Nanoeng. 10 (1), 113. 10.1038/s41378-024-00743-z 39166136 PMC11333603

[B14] Guo R. Liu Y. Xu N. Ling G. Zhang P. (2022). Multifunctional nanomedicines for synergistic photodynamic immunotherapy based on tumor immune microenvironment. Eur. J. Pharm. Biopharm. 173, 103–120. 10.1016/j.ejpb.2022.03.002 35283303

[B15] Haghighat F. Kim Y. Sourinejad I. Yu I. J. Johari S. A. (2021). Titanium dioxide nanoparticles affect the toxicity of silver nanoparticles in common carp (*Cyprinus carpio*). Chemosphere 262, 127805. 10.1016/j.chemosphere.2020.127805 32750593

[B16] Hao X. Wang S. Wang L. Li J. Li Y. Liu J. (2024). Exosomes as drug delivery systems in glioma immunotherapy. J. Nanobiotechnology 22 (1), 340. 10.1186/s12951-024-02611-4 38890722 PMC11184820

[B17] Hapuarachchige S. Zhu W. Kato Y. Artemov D. (2014). Bioorthogonal, two-component delivery systems based on antibody and drug-loaded nanocarriers for enhanced internalization of nanotherapeutics. Biomaterials 35 (7), 2346–2354. 10.1016/j.biomaterials.2013.11.075 24342725 PMC4332786

[B18] Hinna A. H. Hupfeld S. Kuntsche J. Brandl M. (2016). The use of asymmetrical flow field-flow fractionation with on-line detection in the study of drug retention within liposomal nanocarriers and drug transfer kinetics. J. Pharm. Biomed. Anal. 124, 157–163. 10.1016/j.jpba.2016.02.037 26950903

[B19] Islam W. Kimura S. Islam R. Harada A. Ono K. Fang J. (2021). EPR-effect enhancers strongly potentiate tumor-targeted delivery of nanomedicines to advanced cancers: further extension to enhancement of the therapeutic effect. J. Personalized Med. 11 (6), 487. 10.3390/jpm11060487 PMC8229906 34071552

[B20] Jakic K. Selc M. Razga F. Nemethova V. Mazancova P. Havel F. (2024). Long-term accumulation, biological effects and toxicity of BSA-coated gold nanoparticles in the mouse liver, spleen, and kidneys. Int. J. Nanomed. 19, 4103–4120. 10.2147/ijn.S443168 PMC11088863 38736658

[B21] Jing G. Li Y. Sun F. Liu Q. Du A. Wang H. (2024). Near-infrared light-activatable upconversion nanoparticle/curcumin hybrid nanodrug: a potent strategy to induce the differentiation and elimination of glioma stem cells. Adv. Compos. Hybrid Mater. 7 (3), 82. 10.1007/s42114-024-00886-7

[B22] Kumar M. Goswami P. Jha A. Manjit M. Satpute A. P. Koch B. (2024). Formulation and evaluation of cetuximab functionalized phospholipid modified nanocrystals of paclitaxel for non-small cell lung cancer therapy. Sci. Rep. 14 (1), 29114. 10.1038/s41598-024-80283-8 39582089 PMC11586409

[B23] Lee Y. D. Yang J. K. Han S. Kim B. R. Shin J. W. Bang J. (2023). Topical methylene blue nanoformulation for the photodynamic therapy of acne vulgaris. Arch. Dermatol Res. 315 (4), 885–893. 10.1007/s00403-022-02464-7 36376760

[B24] Li C. Wang Z. Lei H. Zhang D. (2023a). Recent progress in nanotechnology-based drug carriers for resveratrol delivery. Drug Deliv. 30 (1), 2174206. 10.1080/10717544.2023.2174206 36852655 PMC9980162

[B25] Li H. Xiao W. Tian Z. Liu Z. Shi L. Wang Y. (2023b). Reaction mechanism of nanomedicine based on porphyrin skeleton and its application prospects. Photodiagnosis Photodyn. Ther. 41, 103236. 10.1016/j.pdpdt.2022.103236 36494023

[B26] Li J. Yi H. Fu Y. Zhuang J. Zhan Z. Guo L. (2025). Biodegradable iridium coordinated nanodrugs potentiate photodynamic therapy and immunotherapy of lung cancer. J. Colloid Interface Sci. 680, 9–24. 10.1016/j.jcis.2024.10.156 39488900

[B27] Li L. Ni R. Zheng D. Chen L. (2023c). Eradicating the tumor “seeds”: nanomedicines-based therapies against cancer stem cells. Daru 31 (1), 83–94. 10.1007/s40199-023-00456-0 36971921 PMC10238364

[B28] Li M. Bian X. Chen X. Fan N. Zou H. Bao Y. (2022a). Multifunctional liposome for photoacoustic/ultrasound imaging-guided chemo/photothermal retinoblastoma therapy. Drug Deliv. 29 (1), 519–533. 10.1080/10717544.2022.2032876 35156504 PMC8863383

[B29] Li S. Zou Q. Li Y. Yuan C. Xing R. Yan X. (2018). Smart peptide-based supramolecular photodynamic metallo-nanodrugs designed by multicomponent coordination self-assembly. J. Am. Chem. Soc. 140 (34), 10794–10802. 10.1021/jacs.8b04912 30102029

[B30] Li W. Xin H. Zhang Y. Feng C. Li Q. Kong D. (2022b). NIR-II fluorescence imaging-guided oxygen self-sufficient nano-platform for precise enhanced photodynamic therapy. Small 18 (51), e2205647. 10.1002/smll.202205647 36328734

[B31] Li Z. Li S. Guo Y. Yuan C. Yan X. Schanze K. S. (2021). Metal-free nanoassemblies of water-soluble photosensitizer and adenosine triphosphate for efficient and precise photodynamic cancer therapy. Acs Nano 15 (3), 4979–4988. 10.1021/acsnano.0c09913 33709690

[B32] Liang P. Mei R. Zongsuo L. (2022). Variation of photosynthesis, secondary metabolites and antioxidant activities in third generation of spaceflight-induced Salvia miltiorrhiza. Chin. Herb. Med. (CHM) 14 (4), 592–601. 10.1016/j.chmed.2022.02.005 36405058 PMC9669357

[B33] Liang S. Le Q. V. Arrua R. D. Turnbull T. Kempson I. (2025). Improved control of triple-negative breast cancer tumor and metastasis with a pH-sensitive hyaluronic acid nanocarrier for doxorubicin delivery. ACS Biomater. Sci. Eng. 11 (1), 623–633. 10.1021/acsbiomaterials.4c01485 39731574

[B34] Lin B. Lin P. Zhang X. Liao Y. Yu Y. Xu X. (2024). Carrier-free, hyaluronic acid-modified self-assembled doxorubicin, and chlorin e6 nanoparticles enhance combined chemo- and photodynamic therapy *in vivo* . Int. J. Nanomed. 19, 14105–14124. 10.2147/ijn.S490485 PMC11700881 39764187

[B35] Liu H. Mei Y. Zhao Q. Zhang A. Tang L. Gao H. (2021). Black phosphorus, an emerging versatile nanoplatform for cancer immunotherapy. Pharmaceutics 13 (9), 1344. 10.3390/pharmaceutics13091344 34575419 PMC8466662

[B36] Liu J. Lyu Q. Wu M. Zhou Y. Wang T. Zhang Y. (2024a). Integrating mTOR inhibition and photodynamic therapy based on carrier-free nanodrugs for breast cancer immunotherapy. Adv. Healthc. Mater 13 (31), e2402357. 10.1002/adhm.202402357 39235716 PMC11650419

[B37] Liu Q. Chen X. Jiang Y. Yan M. Yu B. Zhang W. (2024b). Self-promoted targeting delivery of nanodrug through chemotherapeutic upregulation of CD47 for triple negative breast cancer therapy. Adv. Funct. Mater. 34 (13). 10.1002/adfm.202311677

[B38] Liu W. Nie F. Jiang H. Zhao Y. Zhang Y. Zhang Z. (2024c). Preparation of pH-sensitive polysaccharide-small molecule nanoparticles and their applications for tumor chemo- and immunotherapy. ACS Appl. Mater Interfaces 16 (49), 68437–68452. 10.1021/acsami.4c16504 39586061

[B39] Liu X. Wang H. Li Z. Li J. He S. Hu C. (2024d). Transformable self-delivered supramolecular nanomaterials combined with anti-PD-1 antibodies alleviate tumor immunosuppression to treat breast cancer with bone metastasis. J. Nanobiotechnol. 22 (1), 566. 10.1186/s12951-024-02839-0 PMC11401275 39272206

[B40] Liu Y. Han Y. Chen S. Liu J. Wang D. Huang Y. (2022). Liposome-based multifunctional nanoplatform as effective therapeutics for the treatment of retinoblastoma. Acta Pharm. Sin. B 12 (6), 2731–2739. 10.1016/j.apsb.2021.10.009 35755292 PMC9214327

[B41] Lokesh B. S. Ajmeera S. Choudhary R. Moharana S. K. Purohit C. S. Konkimalla V. B. (2024). Engineering of redox-triggered polymeric lipid hybrid nanocarriers for selective drug delivery to cancer cells. J. Mater Chem. B 13, 1437–1458. 10.1039/d4tb01236d 39690942

[B42] Marques C. Fernandes M. H. Lima S. A. C. (2023). Elucidating berberine's therapeutic and photosensitizer potential through nanomedicine tools. Pharmaceutics 15 (9), 2282. 10.3390/pharmaceutics15092282 37765251 PMC10535601

[B43] Mesquita B. Singh A. Prats Masdeu C. Lokhorst N. Hebels E. R. van Steenbergen M. (2024). Nanobody-mediated targeting of zinc phthalocyanine with polymer micelles as nanocarriers. Int. J. Pharm. 655, 124004. 10.1016/j.ijpharm.2024.124004 38492899

[B44] Naser I. H. Zaid M. Ali E. Jabar H. I. Mustafa A. N. Alubiady M. H. S. (2024). Unveiling innovative therapeutic strategies and future trajectories on stimuli-responsive drug delivery systems for targeted treatment of breast carcinoma. Naunyn Schmiedeb. Arch. Pharmacol. 397 (6), 3747–3770. 10.1007/s00210-023-02885-9 38095649

[B45] Nirmal G. R. Lin Z. C. Lin C. H. Sung C. T. Liao C. C. Fang J. Y. (2022). Polydopamine/IR820 nanoparticles as topical phototheranostics for inhibiting psoriasiform lesions through dual photothermal and photodynamic treatments. Biomater. Sci. 10 (21), 6172–6189. 10.1039/d2bm00835a 36073349

[B46] Qi T. Chen B. Wang Z. Du H. Liu D. Yin Q. (2019). A pH-Activatable nanoparticle for dual-stage precisely mitochondria-targeted photodynamic anticancer therapy. Biomaterials 213, 119219. 10.1016/j.biomaterials.2019.05.030 31132647

[B47] Resende P. V. S. Gomes I. N. F. Peixoto M. C. Stringhetta G. R. Arantes L. Kuzmin V. A. (2025). Evaluation of the antineoplastic properties of the photosensitizer biscyanine in 2D and 3D tumor cell models and artificial skin models. J. Photochem Photobiol. B 262, 113078. 10.1016/j.jphotobiol.2024.113078 39671777

[B48] Sarbadhikary P. George B. P. Abrahamse H. (2022). Potential application of photosensitizers with high-Z elements for synergic cancer therapy. Front. Pharmacol. 13, 921729. 10.3389/fphar.2022.921729 35837287 PMC9274123

[B49] Shi E. Shan T. Wang H. Mao L. Liang Y. Cao M. (2023). A bacterial nanomedicine combines photodynamic-immunotherapy and chemotherapy for enhanced treatment of oral squamous cell carcinoma. Small 19 (52), e2304014. 10.1002/smll.202304014 37653616

[B50] Shi X.-H. Fu D.-D. Wang J.-M. Li J. Ye Q.-Q. Wang Z.-G. (2024). GSH-responsive prodrug-based nanodrugs for augmenting chemo-photodynamic synergistic therapy against tumors. Nano Today 57, 102368. 10.1016/j.nantod.2024.102368

[B51] Sulaiman C. George B. P. Balachandran I. Abrahamse H. (2022). Photoactive herbal compounds: a green approach to photodynamic therapy. Molecules 27 (16), 5084. 10.3390/molecules27165084 36014325 PMC9413332

[B52] Sun L. Zuo C. Ma B. Liu X. Guo Y. Wang X. (2025). Intratumoral injection of two dosage forms of paclitaxel nanoparticles combined with photothermal therapy for breast cancer. Chin. Herb. Med. (CHM) 17 (1), 156–165. 10.1016/j.chmed.2024.06.001 39949814 PMC11814247

[B53] Tang P. Xing M. Xing X. Tao Q. Cheng W. Liu S. (2021). Receptor-mediated photothermal/photodynamic synergistic anticancer nanodrugs with SERS tracing function. Colloids Surfaces B-Biointerfaces 199, 111550. 10.1016/j.colsurfb.2020.111550 33385819

[B54] Wang D. Yi H. Geng S. Jiang C. Liu J. Duan J. (2023a). Photoactivated DNA nanodrugs damage mitochondria to improve gene therapy for reversing chemoresistance. Acs Nano 17 (17), 16923–16934. 10.1021/acsnano.3c04002 37606317

[B55] Wang N. Liu Y. Peng D. Zhang Q. Zhang Z. Xu L. (2024). Copper-based composites nanoparticles improve triple-negative breast cancer treatment with induction of apoptosis-cuproptosis and immune activation. Adv. Healthc. Mater 13 (28), e2401646. 10.1002/adhm.202401646 39001628

[B56] Wang N. Wang Y. Shi R. Lin Y. Jiang X. Feng Y. (2022). The photodynamic/photothermal synergistic therapeutic effect of BODIPY-I-35 liposomes with urea. Photodiagnosis Photodyn. Ther. 37, 102723. 10.1016/j.pdpdt.2022.102723 35032702

[B57] Wang X. Li Y. Hasrat K. Yang L. Qi Z. (2023b). Sequence-responsive multifunctional supramolecular nanomicelles act on the regression of TNBC and its lung metastasis via synergic pyroptosis-mediated immune activation. Small 19 (50), e2305101. 10.1002/smll.202305101 37635105

[B58] Xiao Y. Zhang T. Ma X. Yang Q. C. Yang L. L. Yang S. C. (2021). Microenvironment-responsive prodrug-induced pyroptosis boosts cancer immunotherapy. Adv. Sci. (Weinh) 8 (24), e2101840. 10.1002/advs.202101840 34705343 PMC8693073

[B59] Yan B. Li Y. He S. (2024). Aptamer-mediated therapeutic strategies provide a potential approach for cancer. Int. Immunopharmacol. 136, 112356. 10.1016/j.intimp.2024.112356 38820957

[B60] Yang L. Song S. Yin M. Yang M. Yan D. Wan X. (2023). Antibiotic-based small molecular micelles combined with photodynamic therapy for bacterial infections. Asian J. Pharm. Sci. 18 (3), 100810. 10.1016/j.ajps.2023.100810 37274927 PMC10236462

[B61] Yu T.-T. Han N. Li L.-G. Peng X.-C. Li Q.-R. Xu H.-Z. (2022). Chlorin e6-Induced photodynamic effect polarizes the macrophage into an M1 phenotype through oxidative DNA damage and activation of STING. Front. Pharmacol. 13, 837784. 10.3389/fphar.2022.837784 35308251 PMC8927874

[B62] Yu Y. Yu R. Wang N. Bai Y. Shi Q. Maswikiti E. P. (2023). Photodynamic therapy in combination with immune checkpoint inhibitors plus chemotherapy for first-line treatment in advanced or metastatic gastric or gastroesophageal junction cancer: a phase 2–3 clinical trial protocol. Front. Pharmacol. 14, 1063775. 10.3389/fphar.2023.1063775 36778024 PMC9908746

[B63] Zhang J. Li W. Tao Z. Zhou X. Chen X. Zhou J. (2024). Endogenous glucose-driven cascade reaction of nano-drug delivery for boosting multidrug-resistant bacteria-infected diabetic wound healing. J. Colloid Interface Sci. 672, 63–74. 10.1016/j.jcis.2024.05.204 38830319

[B64] Zhang J. Liang Y. C. Lin X. Zhu X. Yan L. Li S. (2015). Self-monitoring and self-delivery of photosensitizer-doped nanoparticles for highly effective combination cancer therapy *in vitro* and *in vivo* . Acs Nano 9 (10), 9741–9756. 10.1021/acsnano.5b02513 26390118

[B65] Zhang Y. Yang N. Dong Z. Wu J. Liao R. Zhang Y. (2023). Dual-targeting biomimetic nanomaterials for photo-/chemo-/antiangiogenic synergistic therapy. ACS Appl. Mater Interfaces 15 (28), 33288–33298. 10.1021/acsami.3c03471 37400422

[B66] Zhao G. Lu G. Fan H. Wei L. Yu Q. Li M. (2024a). Herbal products-powered thermosensitive hydrogel with phototherapy and microenvironment reconstruction for accelerating multidrug-resistant bacteria-infected wound healing. Adv. Healthc. Mater 13 (15), e2400049. 10.1002/adhm.202400049 38416676

[B67] Zhao L. Chang F. Tong Y. Yin J. Xu J. Li H. (2024b). A multifunctional bimetallic nanoplatform for synergic local hyperthermia and chemotherapy targeting HER2-positive breast cancer. Adv. Sci. (Weinh) 11 (16), e2308316. 10.1002/advs.202308316 38380506 PMC11040336

[B68] Zhao L. P. Rao X. N. Zheng R. R. Huang C. Y. Kong R. J. Cheng H. (2023). Carrier-free nano-PROTACs to amplify photodynamic therapy induced DNA damage through BRD4 degradation. Nano Lett. 23 (13), 6193–6201. 10.1021/acs.nanolett.3c01812 37387510

[B69] Zhao Q. Wang T. Wang H. Cao P. Jiang C. Qiao H. (2024c). Consensus statement on research and application of Chinese herbal medicine derived extracellular vesicles-like particles (2023 edition). Chin. Herb. Med. (CHM) 16 (1), 3–12. 10.1016/j.chmed.2023.11.002 38375050 PMC10874762

[B70] Zhu Y. Wang Z. Zhao R. Zhou Y. Feng L. Gai S. (2022). Pt decorated Ti_3_C_2_T_x_ mXene with NIR-II light amplified nanozyme catalytic activity for efficient phototheranostics. Acs Nano 16 (2), 3105–3118. 10.1021/acsnano.1c10732 35040328

[B71] Zou L. Zhang Y. Cheraga N. Abodunrin O. D. Qu K. Y. Qiao L. (2023). Chlorin e6 (Ce6)-loaded plaque-specific liposome with enhanced photodynamic therapy effect for atherosclerosis treatment. Talanta 265, 124772. 10.1016/j.talanta.2023.124772 37327664

